# Supplementary material for: Resolving the full spectrum of human genome variation using Linked-Reads
Source: Genome Res. 2019 Apr;29(4):635–45. doi: 10.1101/gr.234443.118 (PMC6442396; doi:10.1101/gr.234443.118)
Supplement: Supplemental Material [file supp_gr.234443.118_Supplemental_longranger-2.2.zip › longranger-2.2.tar.bz2!/lr2.2-public/lib/go/src/code.google.com/vitess/docs/contributing/index.html]

Vitess / Contributing to Vitess


Toggle navigation

- Overview
- Guides
- Reference
- Blog
- GitHub

- #### Overview

  - What is Vitess
  - Scaling MySQL with Vitess
  - Key Concepts
- #### Getting Started

  - Run Vitess on Kubernetes
    - Custom Docker Build
  - Run Vitess Locally
- #### User Guide

  - Introduction- Client Libraries- Backing Up Data- Reparenting
        - Schema Management
        - VSchema Guide
        - Schema Swap (Tutorial)
        - Sharding
          - Horizontal Sharding (Tutorial, automated)
          - Horizontal Sharding (Tutorial, manual)
          - Sharding in Kubernetes (Tutorial, automated)
          - Sharding in Kubernetes (Tutorial, manual)
        - Vitess Sequences
        - MySQL Server Protocol
        - Vitess and Replication
        - Update Stream
        - Row Based Replication
        - Topology Service
        - Transport Security Model
        - Launching
          - Scalability Philosophy
          - Production Planning
          - Server Configuration
          - 2PC Guide
          - Troubleshooting
        - Upgrading
- #### Reference Guides

  - Vitess API- vtctl Commands
- #### Other Resources

  - Presentations- Blog- Roadmap
- #### Contributing

  - Contributing to Vitess- GitHub Workflow- Code Reviews
- GitHub

# Contributing to Vitess

- #### Overview

  - What is Vitess
  - Scaling MySQL with Vitess
  - Key Concepts
- #### Getting Started

  - Run Vitess on Kubernetes
    - Custom Docker Build
  - Run Vitess Locally
- #### User Guide

  - Introduction- Client Libraries- Backing Up Data- Reparenting
        - Schema Management
        - VSchema Guide
        - Schema Swap (Tutorial)
        - Sharding
          - Horizontal Sharding (Tutorial, automated)
          - Horizontal Sharding (Tutorial, manual)
          - Sharding in Kubernetes (Tutorial, automated)
          - Sharding in Kubernetes (Tutorial, manual)
        - Vitess Sequences
        - MySQL Server Protocol
        - Vitess and Replication
        - Update Stream
        - Row Based Replication
        - Topology Service
        - Transport Security Model
        - Launching
          - Scalability Philosophy
          - Production Planning
          - Server Configuration
          - 2PC Guide
          - Troubleshooting
        - Upgrading
- #### Reference Guides

  - Vitess API- vtctl Commands
- #### Other Resources

  - Presentations- Blog- Roadmap
- #### Contributing

  - Contributing to Vitess- GitHub Workflow- Code Reviews

# Contributing to Vitess

You want to contribute to Vitess? That's awesome!

In the past we have reviewed and accepted many external contributions. Examples are the Java JDBC driver, the PHP PDO driver or vtgate v3 improvements.

We're looking forward to any contribution! Before you start larger contributions, make sure to reach out first and discuss your plans with us.

This page describes for new contributors how to make yourself familiar with Vitess and the programming language Go.

## Learning Go

Vitess was one of the early adaptors of Google's programming language Go.

We love it for its simplicity (e.g. compared to C++ or Java) and performance (e.g. compared to Python).

Contributing to our server code will require you to learn Go. We recommend to read the following resources.

### Go Tour

https://tour.golang.org/

The Go tour is a browser based tutorial which explains the different concepts of the programming language.
It's interactive i.e. you can change and run all examples on the right side.
The later steps also have specific exercises which you're supposed to implement yourself.
It's a lot of fun and demonstrates how simple it is to write Go code.

### Go Readability

Internally at Google, code reviews are subject to an additional "readability" review.

A readability reviewer ensures that the reviewee is writing idiomatic code and following the programming language's style guide.

While there's no Go style guide, there is a set of recommendations in the Go community which add up to an implicit style guide.
To make sure you're writing idiomatic Go code, please read the following documents:

- Go Readablity slides: https://talks.golang.org/2014/readability.slide
  - Talk about Go readability with many specific examples.
- "Effective Go": https://golang.org/doc/effective\_go.html
  - Recommendations for writing good Go code.
- Go Code Review Comments: https://github.com/golang/go/wiki/CodeReviewComments
  - The closest thing to a style guide.

### Other Resources

If you're unsure about Go's behavior or syntax, we recommend to look it up in the specification: https://golang.org/ref/spec
It is well written and easy to understand.

### Appreciating Go

After using Go for several weeks, we hope that you'll start to love Go as much as we do.

In our opinion, the song "Write in Go" from ScaleAbility, a Google acapella band, perfectly captures what's so special about Go. Watch it and enjoy that you learnt Go: www.youtube.com/watch?v=LJvEIjRBSDA

## Learning Vitess

Before diving into the Vitess codebase, make yourself familiar with the system and run it yourself:

- Read the What is Vitess page, in particular the architecture section.
- Read the Vitess concepts and the Sharding page.

  - We also recommend to look at our latest presentations. They contain many illustrations which help understanding how Vitess works in detail.
  - After studying the pages, try to answer the following question (click expand to see the answer):

    Let's assume a keyspace with 256 range-based shards: What is the name of the first, the second and the last shard?
    -01, 01-02, ff-
- Go through the Vitess Kubernetes tutorial.

  - While going through the tutorial, look back at the architecture and match the processes you start in Kubernetes with the boxes in the diagram.

Contact: vitess@googlegroups.com  **·**  
Announcements  **·**  
© 2017 Vitess powered by Google Inc
